# Value of N1 Lymph Node Examination in the Prognosis of Patients With pT1-3N0M0 Non-Small Cell Lung Cancer

**DOI:** 10.3389/fonc.2020.603378

**Published:** 2020-12-15

**Authors:** Gaoxiang Wang, Xianning Wu, Xiaohui Sun, Tian Li, Meiqing Xu, Liangdong Xu, Mingran Xie

**Affiliations:** ^1^ Department of Thoracic Surgery, Affiliated Provincial Hospital of Anhui Medical University, Hefei, China; ^2^ Department of Thoracic Surgery, The First Affiliated Hospital of USTC, Division of Life Sciences and Medicine, University of Science and Technology of China, Hefei, China

**Keywords:** examined lymph node, non-small cell lung cancer, survival, prognosis, surgery

## Abstract

**Objective:**

This study aimed to analyze the relationship between the number of examined lymph nodes (ELNs) at the N1 station and the postoperative clinicopathological features and prognosis of patients with pT1-3N0M0 non-small cell lung cancer (NSCLC).

**Methods:**

The cut-off value of the number of ELNs at the N1 station was obtained using X-tile software analysis. Kaplan-Meier survival curve analysis and the Cox proportional hazard model were used to study the impact of the number of ELNs at the N1 station on the prognosis of postoperative patients with pT1-3N0M0 NSCLC.

**Results:**

The median survival time and 1-, 3- and 5-year survival rates of 0 ELNs at the N1 station were 28.0 months and 74.8%, 45.4%, and 21.2%, respectively. The median survival time and 1-, 3-, and 5-year survival rates of 1–4 ELNs at the N1 station were 45.0 months and 85.5%, 55.4%, and 39.1%, respectively. In the group with ≥ 5 ELNs at the N1 station, the median survival time and the 1-, 3- and 5-year survival rates were 59.0 months and 94.0%, 62.7%, and 48.2%, respectively. Both univariate and multivariate Cox regression analyses showed that the number of ELNs at the N1 station, T stage and operation type were independent factors affecting the prognosis of patients with pT1-3N0M0 NSCLC.

**Conclusion:**

Increasing the number of ELNs at the N1 station is positively correlated with the long-term survival rate of patients with T1-3N0M0 NSCLC. At least 5 LNs at the N1 station should be examined in pathological examination.

## Introduction

The morbidity and mortality of lung cancer are ranked first among all malignant tumors in the world (1, 2). The lung cancer histological types small cell lung cancer and non-small cell lung cancer (NSCLC) comprise 85% of all lung cancers, of which NSCLC accounts for 80% (1, 3). Radical resection remains the primary treatment for early resectable NSCLC. However, the postoperative 5-year survival rate is only 50% to 60% (4).

NSCLC patients with positive lymph nodes have a high risk of recurrence after surgery. Lymph node involvement is an important factor that determines the prognosis and treatment decision of postoperative NSCLC patients. Lymph node resection or sampling plays a crucial role in accurate lymph node staging. The results of lymph node pathological examination can determine the postoperative lymph node stage of the patient, determine the postoperative pathological stage of the patient, and guide the next step of treatment. Accurate postoperative staging is the key to determine whether patients should receive adjuvant therapy after surgery. Additionally, according to the International Association for Lung Cancer Research’s lymph node map, the NCCN guidelines recommend that thoracic surgeons sample only one or more lymph nodes from mediastinal lymph node stations (2R, 4R, 7, 8 and 9 on the right; 4L, 5, 6, 7, 8 and 9 on the left) (5, 6).

Because lymph nodes at the N1 station (stations 10, 11, 12, 13 and 14) are located in the lungs, they are often not examined by surgeons and pathologists after surgery. Thus, postoperative pathological N1 stage is evaluated as N0, resulting in the lack of adjuvant treatment after an operation and increasing the risk of postoperative recurrence. Presently, many studies have reported on the number of examined lymph nodes (ELNs) in patients with resectable NSCLC (7–9), but few have focused on the minimum number of ELNs at the N1 station. Therefore, this study aimed to explore whether the number of ELNs at the N1 station affects the long-term survival of patients with pT1-3N0M0 NSCLC.

## Methods

### Patient Selection

This study evaluated 1,510 NSCLC patients who had undergone radical resection of lung cancer at the affiliated Provincial Hospital of Anhui Medical University between January 2010 and April 2015. All the patients received lobectomy (or pneumonectomy) plus systematic hilar and mediastinal lymph node dissection. The inclusion criteria were as follows: 1) pathological diagnosis of NSCLC; 2) postoperative pathological stage of pT1-3N0M0; and 3) R0 resection. The exclusion criteria were as follows: 1) distant metastasis found during surgery or confirmed to be T4N1-2M1, 2) neoadjuvant therapy before surgery, and 3) incomplete medical records.

The clinical data were obtained from the LinkDoc database, supplemented by electronic medical record review. In this study, TNM staging, T staging, N staging and M staging were performed according to the 8th edition of the Lung Cancer Staging Project of the American Joint Commission on Cancer (AJCC). This study was approved by the Affiliated Provincial Hospital of Anhui Medical University.

### Variables and Outcomes

This study collected and analyzed sex, age, smoking history, operation type, tumor location, tumor diameter, histology, tumor differentiation, T stage, preoperative complications and postoperative chemotherapy in patients with T1-3N0M0 NSCLC. The primary endpoint was overall survival (OS), which was defined as the interval between the date of operation and date of death from any cause or the last follow-up. We surveyed the patients at 3-month intervals for the first 2 years and at 6-month intervals thereafter. The follow-up evaluations generally included a physical examination, blood analysis (including pertinent tumor markers), chest radiography, and chest CT. Whenever there were symptoms or signs indicative of recurrence, further evaluations, including CT of the abdomen, brain magnetic resonance imaging, positron emission tomography-CT or bone scintigraphy, were performed.

### Statistical Analyses

The categorical and continuous variables between the groups were compared by χ2-test and independent samples t-test as appropriate. The optimal number of ELNs at the N1 station was calculated using the X-tile model based on the maximum chi-squared value when a series of log-rank tests were conducted. Survival curves were depicted by the Kaplan-Meier method and compared among groups using the log-rank test. Multivariate Cox regression analyses were applied to identify the independent predictors for survival. Statistical analyses were conducted using SPSS Statistics (version 24; IBM, NY, USA). Differences with p < 0.05 were considered statistically significant.

## Results

### Patient Characteristics

In total, 702 patients were included in this study. According to the relationship between the number of ELNs at the N1 station and OS in patients with pT1-3N0M0 NSCLC, the cohort was divided into three groups based on the X-tile model: 0 ELNs at the N1 station, 1–4 ELNs at the N1 station and ≥ 5 ELNs at the N1 station. 0 ELNs at the N1 station were defined as Group A, 1–4 ELNs at the N1 station were defined as Group B, and ≥ 5 ELNs at the N1 station were defined as Group C. The baseline characteristics are depicted in **Table 1**. The T stage had a significant influence on the number of ELNs at the N1 station. The results showed that the later was the T stage, the greater was the effect on the number of ELNs at the N1 station. Additionally, no significant difference was found in age, sex, smoking history, operation type, tumor location, tumor diameter, histology, tumor differentiation, preoperative complications or postoperative chemotherapy among the three groups.

**Table 1:** Baseline characteristics of the study population.

	Group A (n = 152)	Group B (n = 281)	Group C (n = 269)	χ*^2^*	*P Value*
**Sex**				3.770	0.152
Male	110(72.4%)	185(65.8%)	196(72.9%)		
Female	42(27.6%)	96(34.2%)	73(27.1%)		
**Age,years**				0.427	0.808
≤ 60	69(45.4%)	133(47.3%)	131(48.7%)		
>60	83(54.6%)	148(52.7%)	138(51.3%)		
**Smoking history**				4.168	0.124
Yes	48(31.6%)	107(38.1%)	112(41.6%)		
No	104(68.4%)	174(61.9%)	157(58.4%)		
**Operation type**				2.566	0.277
Lobectomy	136(89.5%)	261(92.9%)	240(89.2%)		
Pneumonectomy	16(10.5%)	20(7.1%)	29(10.8%)		
**Tumor location**				11.942	0.154
RUL	46(30.3%)	68(24.2%)	66(24.5%)		
RML	13(8.6%)	30(10.7%)	17(6.3%)		
RLL	30(19.7%)	50(17.8%)	61(22.7%)		
LUL	42(27.6%)	87(31.0%)	67(24.9%)		
LLL	21(13.8%)	46(16.4%)	58(21.%)		
**Tumor diameter, cm**				2.412	0.299
≤3	63(41.4%)	96(34.2%)	103(37.3%)		
>3	89(58.6%)	185(65.8%)	166(62.7%)		
**Histology**				2.641	0.619
ADC	89(58.6%)	166(59.1%)	144(53.5%)		
SCC	52(34.2%)	100(35.6%)	108(40.1%)		
Other*	11(7.2%)	15(5.3%)	17(6.3%)		
**Tumor differentiation**				9.380	0.052
High	6(3.9%)	10(3.6%)	11(4.1%)		
Medium	41(27.0%)	114(40.6%)	108(40.1%)		
Low	105(69.1%)	157(55.9%)	150(55.8%)		
**T stage**				16.876	0.002
T1	40(26.3%)	96(34.2%)	53(19.7%)		
T2	71(46.7%)	120(42.7%)	152(56.5%)		
T3	41(27.0%)	65(23.1%)	64(23.8%)		
**Postoperative chemotherapy**				2.570	0.277
Yes	35(9.9%)	16(5.7%)	20(7.4%)		
No	117(90.1%)	265(94.3%)	249(92.6%)		
Preoperative Complications*				1.864	0.394
Yes	58(38.2%)	96(34.2%)	85(31.6%)		
No	94(61.8%)	185(65.8%)	184(68.4%)		

RUL, right upper lung; RML, Right middle lung; RLL, Right lower lung; LUL, Left upper lung; LLL, Left lower lung; ADC, adenocarcinoma; SCC, squamous cell carcinoma. * Includes adenosquamous carcinoma, giant-cell carcinoma, sarcoma, and so forth. *Includes high blood pressure, diabetes, arrhythmia, and so forth.

**Figure 1:**
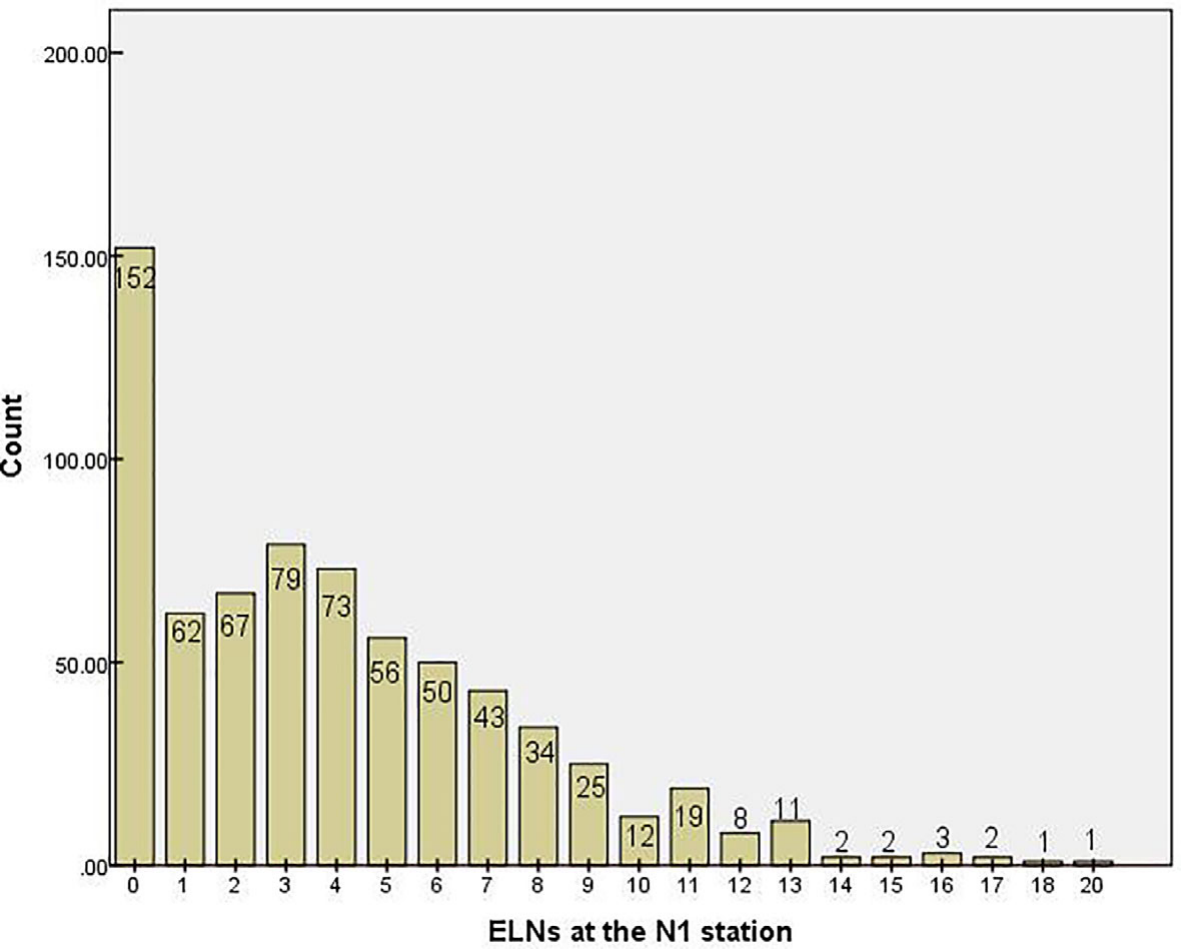
Distribution of the number of examined lymph nodes at the N1 stationin. ELN, examined lymph node.

In total, 9,608 lymph nodes were examined in the whole group, of which the number of ELNs at the N1 station was 2,849. The median number of ELNs was 13 (range: 0–35), and the median number of stations at which lymph nodes were examined was 3 (range: 0–8). The median number of ELNs at the N1 station was 3 (range: 0–20). The distribution of the number of ELNs at the N1 station in the NSCLC patients is shown in **Figure 1**.

### Impact of the Number of Examined Lymph Nodes on Survival

**Figure 2:**
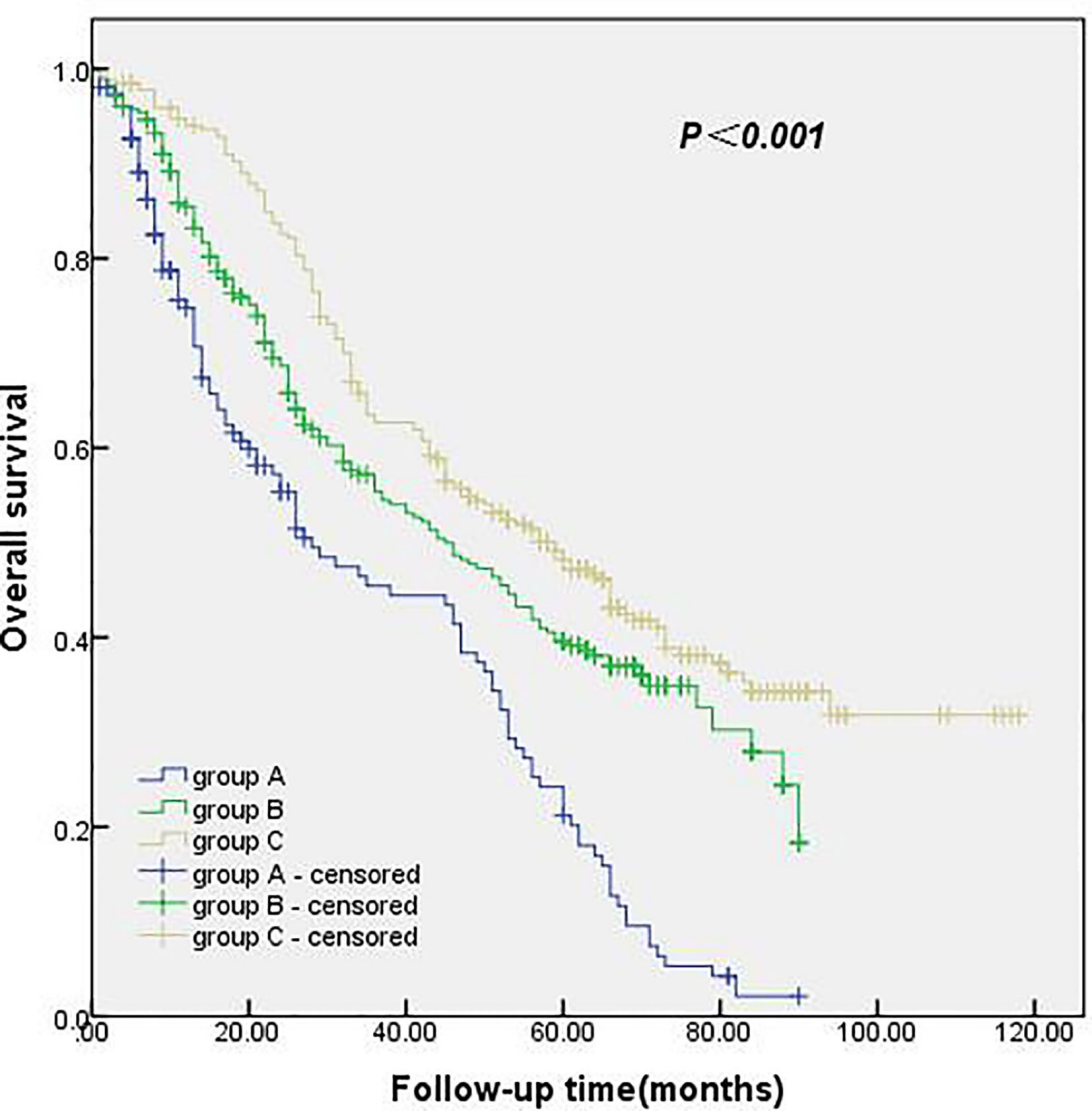
Kaplan-Meier survival curves for group A, B and C.

The follow-up period was from January 2010 to April 2020, with a total follow-up period of 112 months. The median survival time and 1-, 3- and 5-year survival rates were 46.0 months and 86.6%, 56.1%, and 39.5%, respectively. The median survival time and 1-, 3- and 5-year survival rates of 0 ELNs at the N1 station were 28.0 months and 74.8%, 45.4%, and 21.2%, respectively. The median survival time and 1-, 3-, and 5-year survival rates of 1–4 ELNs at the N1 station were 45.0 months and 85.5%, 55.4%, and 39.1%, respectively. In the group with ≥ 5 ELNs at the N1 station, the median survival time and 1-, 3- and 5-year survival rates were 59.0 months and 94.0%, 62.7%, and 48.2%, respectively. The survival rate of the group with ≥ 5 ELNs at the N1 station was significantly better than that of the group with 0 ELNs at the N1 station and the group with 1–4 ELNs at the N1 station. These results were statistically significant (*p*<0.001). The details are shown in **Figure 2**.

Univariate analysis of the clinicopathological data showed that the operation type, histology, T stage and ELNs at the N1 station were significantly correlated with patient survival (*p*<0.05). The details are shown in **Table 2**. The clinicopathological data of the patients were incorporated into the Cox model for multivariate analysis. The results showed that the operation type, T stage and ELNs at the N1 station were independent prognostic factors affecting patient survival (p<0.05). The details are shown in **Table 3**.

**Table 2:** The prognostic factors associated with overall survival of patients in groups A, B, and C by univariate analysis.

	Case	Median survival time (month) (95% CI)	5-year survival rate (%)	*P Value*
**Sex**				0.430
Male	491	47.0 ± 3.590	41.5	
Female	211	46.0 ± 5.318	35.5	
**Age,years**				0.366
≤ 60	333	44.0 ± 4.806	37.5	
>60	369	49.0 ± 3.216	41.3	
**Smoking history**				0.843
Yes	267	45.0 ± 5.471	40.0	
No	435	49.0 ± 2.767	39.2	
**Operation type**				0.006
Lobectomy	637	48.0 ± 2.515	40.7	
Pneumonectomy	65	29.0 ± 5.459	28.0	
**Tumor location**				0.134
RUL	180	43.0 ± 4.900	37.0	
RML	60	54.0 ± 7.916	42.0	
RLL	141	56.0 ± 5.012	44.7	
LUL	196	42.0 ± 5.519	36.6	
LLL	125	47.0 ± 4.148	39.8	
**Tumor diameter, cm**				0.150
≤3	262	50.0 ± 5.033	42.8	
>3	440	45.0 ± 3.347	37.3	
**Histology**				0.010
ADC	399	51.0 ± 2.924	40.6	
SCC	260	45.0 ± 4.597	40.9	
Other*	43	31.0 ± 5.423	22.7	
**Tumor differentiation**				0.546
High	27	62.0 ± 5.639	51.5	
Medium	263	47.0 ± 4.522	38.3	
Low	412	45.0 ± 4.064	38.8	
**T stage**				0.008
T1	189	54.0 ± 5.235	44.7	
T2	343	50.0 ± 3.781	41.1	
T3	170	35.0 ± 2.505	29.6	
**Postoperative chemotherapy**				0.510
Yes	51	43.0 ± 6.763	31.3	
No	651	47.0 ± 2.697	40.1	
Preoperative complications*				0.676
Yes	239	45.0 ± 3.501	39.2	
No	463	53.0 ± 3.648	40.2	
**ELNs at N1 station**				<0.001
0	152	28.0 ± 5.863	21.2	
1-4	281	45.0 ± 5.051	39.1	
≥5	269	59.0 ± 4.772	48.2	

RUL, right upper lung; RML, right middle lung; RLL, right lower lung; LUL, left upper lung; LLL, left lower lung; ADC, adenocarcinoma; SCC, squamous cell carcinoma. *Includes adenosquamous carcinoma, giant-cell carcinoma, sarcoma, and so forth. ELNs, examined lymph node. *Includes high blood pressure, diabetes, arrhythmia, and so forth.

**Table 3:** The prognostic factors associated with overall survival of patients in groups A, B, and C by multivariate Cox regression.

	*P Value*	RR (95% CI)
**ELNs at N1 station**	<0.001	0.638(0.562–0.725)
**Operation type**	0.025	1.429(1.046–1.951)
**T stage**	0.032	1.166(1.013–1.341)
**Sex**	0.186	–
**Age**	0.264	–
**Smoking history**	0.939	–
**Tumor location**	0.961	–
**Tumor diameter**	0.463	–
**Histology**	0.483	–
**Tumor differentiation**	0.497	–
**Postoperative chemotherapy**	0.792	–
Preoperative complications*	0.413	–

ELNs, examined lymph nodes. *Includes high blood pressure, diabetes, arrhythmia, and so forth.

### Impact of the Number of Examined Lymph Nodes on T Stage

**Figure 3:**
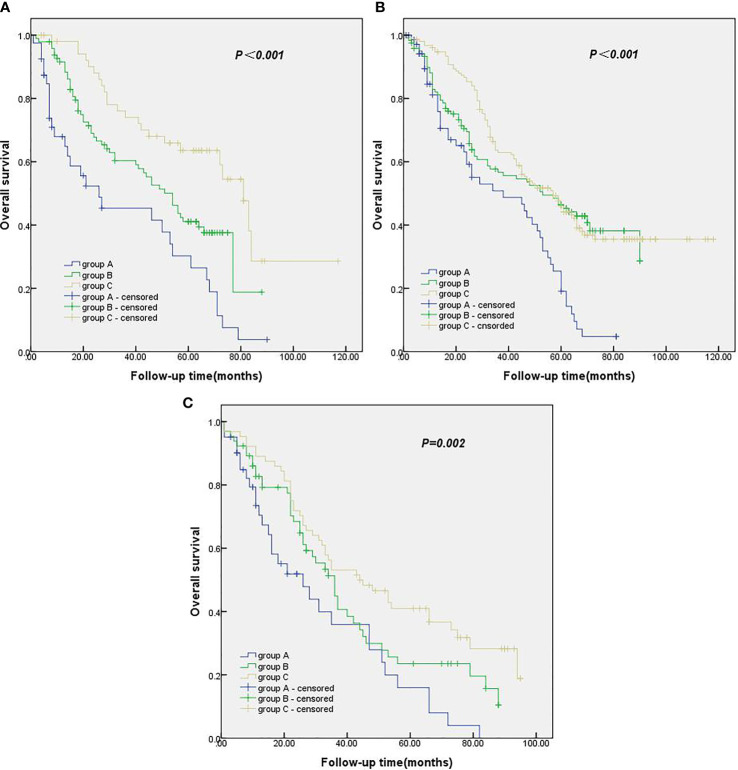
**(A)** Kaplan-Meier survival curves of groups A, B and C in T1 stage. **(B)** Kaplan-Meier survival curves of groups A, B and C in T2 stage. **(C)** Kaplan-Meier survival curves of groups A, B and C in T3 stage.

In comparing the pathological data, we found a significant difference in T stage among the three groups. Therefore, all the patients were classified according to T stage to test whether the number of ELNs at the N1 station in this study was suitable for patients with different T stages of NSCLC. In patients with stage T1, stage T2 and stage T3 NSCLC, the survival rate of the group with ≥ 5 ELNs at the N1 station was significantly better than that of the group with 0 ELNs at the N1 station and the group with 1–4 ELNs at the N1 station, with a statistically significant difference (**Figures 3A–C**).

## Discussion

The problem of postoperative recurrence in patients with NSCLC has always been a major issue faced by clinicians. The NCCN guidelines suggest that inadequate examination of lymph nodes reduces survival and recommend a minimum number of ELNs. For many cancers, such as gastric and breast cancer, the relationship between more lymph node detection and improved long-term survival has been well studied (10, 11). Additionally, studies have shown that insufficient detection of lymph nodes will increase the risk of recurrence in patients with NSCLC. However, few studies have investigated whether the number of ELNs at the N1 station affects the prognosis of patients with NSCLC (12–14). An accurate examination of lymph nodes has important reference significance for the postoperative pathological staging and prognosis evaluation of patients with NSCLC. In this study, the number of ELNs at the N1 station was significantly correlated with the long-term survival rate of pT1-3N0M0 NSCLC patients. The survival rate of patients with ≥ 5 ELNs at the N1 station was higher than that of the other two groups. Additionally, the survival rate of patients with 1–4 ELNs at the N1 station was higher than that of patients with 0 ELNs at the N1 station.

In this study, pT1-3N0M0 NSCLC patients who had undergone radical resection of lung cancer were divided into groups according to the number of ELNs at the N1 station. Through survival and multivariate analyses, we found that the long-term survival rate of pT1-3N0M0 NSCLC patients with ≥ 5 ELNs at the N1 station was higher than that of patients with 1–4 ELNs at the N1 station and patients with 0 ELNs at the N1 station, and the number of ELNs at the N1 station was an independent prognostic factor. Through analyses of the Chinese registry and Surveillance, Epidemiology, and End Results (SEER) database, Liang et al. found that a greater number of ELNs was related to more accurate lymph node staging and the long-term prognosis of NSCLC patients after surgery (15). Additionally, Liang et al. suggested that 16 lymph nodes should be examined as the best cut-off point for postoperative lymph node examination or prognosis stratification in N0 patients (15). Saynak M et al., by evaluating stage Ia NSCLC patients who had undergone lung cancer surgery, found that the local recurrence rate of patients with N1 station lymph nodes examined was 14%, while the recurrence rate of patients without N1 station lymph nodes examined was 31%, suggesting that insufficient examination of N1 station lymph nodes cannot accurately determine the patient’s pathological stage, resulting in insufficient postoperative adjuvant therapy (16).

We believe that the most important findings from this study are as follows. First, when the number of ELNs at the N1 station is low, the probability of positive hilar and intrapulmonary lymph nodes will be reduced, resulting in some postoperative NSCLC patients being mistakenly classified as N0 and in lower chances of postoperative adjuvant therapy and a worse prognosis. When more lymph node examinations are performed at the N1 station, the postoperative lymph node staging of NSCLC patients is more accurate, the chance of receiving adjuvant therapy is increased, and the prognosis is better. Such differences could explain the different survival times among the three groups. This phenomenon is considered the main reason for the results of this study. Second, the first site of lymph node metastasis in patients with NSCLC is the lymph nodes at the N1 station, and the examination of additional pathological sections of lymph nodes at the N1 station will increase the chance of lymph node micrometastasis at the N1 station being detected by pathologists. Thus, patients with stage N0 NSCLC can be accurately classified as stage N1, thus receiving adjuvant therapy postoperation to prolong survival.

In this study, NSCLC patients were refined according to T stage. Survival analysis showed that, in stages T1, T2 and T3, the long-term survival rate of patients with more than 5 ELNs at the N1 station was higher than that of patients with 1–4 ELNs at the N1 station and of patients with 0 ELNs at the N1 station. According to the results of this study, for patients with stage T1 to T3 NSCLC, thoracic surgeons should examine 5 or more N1 lymph nodes during the operation so that patients can obtain good and accurate postoperative pathological staging and sufficient postoperative adjuvant therapy to improve their prognosis. Additionally, through univariate and multivariate Cox regression, we found that the operation type is an independent prognostic factor for the prognosis of patients with pT1-3N0M0 NSCLC. We believe there are several reasons for this finding. First, 637 patients had undergone lobectomy, accounting for 90.7% of the whole population, whereas the percentage of patients who had undergone pneumonectomy accounted for 9.3%. Therefore, there was a certain statistical deviation. Second, most of the patients who had undergone pneumonectomy had central lung cancer, although they were pT1-3N0M0 patients, but the pathological stage was “later” than that of peripheral lung cancer, their survival time was generally short, and their prognosis was poor. Finally, patients who undergo pneumonectomy are more likely to develop respiratory failure, heart failure and other multiple organ failure than those who undergo lobectomy, thus affecting the prognosis.

Our study has several limitations. The main limitations are the retrospective nature of the analysis and nonrandomization, which may have resulted in potential selection bias. Another limitation is that it is difficult to separate each lymph node in the dissected tissue, leading to varying numbers of ELNs due to the fragmentation of lymph node tissue in the process of lymph node resection and possibly affecting the best cut-off point of the number of ELNs.

## Conclusions

In patients with pT1-3N0M0 NSCLC, the long-term survival rate of those with ≥ 5 ELNs at the N1 station was higher than that of patients with 1–4 ELNs at the N1 station and with 0 ELNs at the N1 station, and the number of ELNs at the N1 station was an independent prognostic factor. T stage and surgical method were identified as independent prognostic factors in patients with pT1-3N0M0 NSCLC. Considering some of the limitations of this study, a large cohort, multicenter and prospective study is needed to confirm our findings.

## Data Availability Statement

The original contributions presented in the study are included in the article/supplementary material, further inquiries can be directed to the corresponding author.

## Ethics Statement

The studies involving human participants were reviewed and approved by the Affiliated Provincial Hospital of Anhui Medical University. Written informed consent for participation was not required for this study in accordance with the national legislation and the institutional requirements.

## Author Contributions

GXW conceptualized the study, contributed to the methodology, provided the software, contributed to the investigation of the study, wrote the original draft, and wrote, reviewed, and edited the manuscript. XNW wrote the original draft, and wrote, reviewed, and edited the manuscript. XHS and TL contributed to the visualization and investigation of the study. MQX and LDX provided the software Software and contributed to the validation of the study. MRX acquired funding, and wrote, reviewed, and edited the manuscript. All authors contributed to the article andapproved the submitted version.

## Funding

This work was supported by the grants from the National Natural Science Foundation of China, The Fundamental Research Funds for the Central Universities and Key research and development projects in Anhui Province (Nos. 81973643, WK9110000021, and 202004j07020017).

## Conflict of Interest

The authors declare that the research was conducted in the absence of any commercial or financial relationships that could be construed as a potential conflict of interest.
